# What about diversity? The effect of organizational economic inequality on the perceived presence of women and ethnic minority groups

**DOI:** 10.1371/journal.pone.0271356

**Published:** 2022-08-17

**Authors:** Eva Moreno-Bella, Clara Kulich, Guillermo B. Willis, Miguel Moya

**Affiliations:** 1 Department of Psychology, University of Córdoba, Córdoba, Spain; 2 Mind, Brain and Behaviour Research Centre (CIMCYC), University of Granada, Granada, Spain; 3 Department of Psychology, University of Geneva, Geneva, Switzerland; University of Almería, SPAIN

## Abstract

Economic inequality shapes the degree to which people and different social groups are perceived in stereotypical ways. Our research sought to investigate the impact of the perception of economic inequality in an organizational setting on expectations of social diversity in the organization’s workforce, across the dimensions of gender and ethnicity. Combining data from previous experiments, we first explored in one set of studies (Studies 1a and 1b; *N* = 378) whether the degree of economic inequality in a fictitious organization affected participants’ expectations of the representation of minority vs. majority group employees. We found that when we presented an organization with unequal (vs. equal) distribution of economic wealth amongst its employees to study participants, they expected the presence of men and White majority individuals to be larger than the presence of women and ethnic minorities. Second, we tested our hypotheses and replicated these initial effects in a pre-registered study (Study 2: *N* = 449). Moreover, we explored the potential mediating role of perceived diversity climate, that is, the perception that the organization promotes and deals well with demographic diversity. Findings revealed that an organizational setting that distributed resources unequally (vs. equally) was associated with a more adverse diversity climate, which, in turn, correlated with expectations of a lower presence of minority group employees in the organization. We concluded that economic inequality creates a context that modulates perceptions of a climate of social exclusion which likely affects the possibilities for members of disadvantaged groups to participate and develop in organizations.

## Introduction

People have a need to construct their social world [1]. To achieve this, they tend to categorize individuals into social groups—along dimensions of gender, ethnicity, or age, among others—because this allows them to organize their knowledge and to explain their actions in relation to social norms and expectations existing in society [2]. Social categorization and the inferences it entails may be influenced by social clues and the societal context in which people make judgments. Economic inequality can be one of these contexts [3], and there is evidence that it can influence social perceptions of people and groups along—for example—the stereotype dimensions of competence and warmth [4], and of masculinity and femininity [5].

The present work has three aims, which will be outlined in the following sections. First, we were interested in looking at the psychological consequences of perceived economic inequality in the specific context of organizations. Second, we sought to understand whether perceived economic inequality would influence perceptions of who, in terms of gender- and ethnicity-related diversity, is expected to work in an organization. And third, we explored one potential mechanism for explaining such expectations. One set of two exploratory studies (Studies 1a and 1b) and one pre-registered hypothesis-testing study (Study 2) examined these aims with an experimental methodology.

### Two related settings: Economic inequality at the societal and organizational level

Economic inequality exists and is increasing in most societies [6, 7]. Indeed, it has been proposed that unequal resource distribution is one of the most relevant factors that influence norms in societies [8]. On an individual level, it increases status anxiety and decreases trust towards other people [9–12], which likely impacts how individuals will interact with others. Indeed, in countries high in economic inequality as measured with the Gini index, people tended to show higher ambivalence of stereotypes [4, 13] and believe competitiveness is higher between individuals [14]. On a micro level, perceptions of economic inequality also have significant psychosocial consequences for individuals [15–19]. The level of perceived economic inequality creates a normative climate from which people infer the nature of the most probable interpersonal and intergroup interactions [20, 21]. Perception of relatively high economic inequality causes individuals to expect a more competitive climate and the priority of exchange relationships [20], a greater “merit gap” between rich and poor people [22], and the stronger presence of masculine normative traits at the expense of more feminine ones [5]. Overall, higher inequality seems to be associated with expectations of a more competitive climate, that is, interactions with other individuals that demand agentic and instrumental behaviours.

The growing research on economic inequality and its effects on the perception of the normative climate and traits has widely focused on a societal level (i.e., the economic differences between social class groups [17]), and less research has focused on an organizational setting [23]. It is necessary to apply the concept of economic inequality to organizational reality because wage inequality between workers contributes to societal economic inequality. In 2020, the highest-ranked employee at one of the top 350 firms in the U.S. earned 351 times more than a medium-ranked employee, and this figure appears to be increasing as general economic inequality does [24]. Earnings and intergroup relations are a defining part of organizations that employ, pay, and coordinate individuals’ actions towards a common goal [25]. The presence of wage inequalities within organizational hierarchies, apart from being a reality for most organizational members’ daily lives, impacts individuals’ psychological processes [26, 27]. For example, recent studies have shown that economic inequality in organizations influences intergroup relations by making wealth-based social categories more salient and by leading to a higher likelihood of the perception of conflicts in the organizations [28]. Hence, organizations are an interesting concrete setting in which to examine the consequences of perceived economic inequality [29, 30], and our first aim was thus to conduct research in this setting.

### Economic inequality and the perception of women and ethnic minority groups as members of the organization

Economic inequality in an organization refers not only to different economic resources and rewards for the same work, but also to the assignment of excessive (economic) value to certain skills, values, and attitudes and very little to others [26]. These deep-level diversity characteristics are often perceived as congruent with characteristics at a surface level [31], meaning the visible or easily detectible demographic attributes (e.g., gender, ethnicity, and age [32–35]) by which individuals categorize people. Thus, not only does placing higher value on certain skills and values lead to the selection and promotion of people based on these deep-level criteria, it simultaneously leads to the inclusion of members of social categories that are stereotypically associated with and expected to display these skills and values—mostly male and White employees—resulting in discrimination of other groups (e.g., women and ethnic minorities).

One could thus ask whether the perception of economic inequality in an organization would decrease the perception of the presence of women and ethnic minority groups. We argue that this should indeed be the case. According to prior literature, people perceive disadvantaged groups (i.e., powerless and low-status groups such as women and ethnic minorities) as more communal (i.e., warm and good-natured) and less agentic (i.e., less competitive and less intelligent) or, in the case of the most stigmatized, as neither communal nor agentic at all [36]. Such tendencies are stronger when the context is economically unequal [4]. Indeed, members of more unequal societies are primarily perceived as being dominant and having agentic attributes, [5] to the detriment of communal attributes [20, 37]. Thus, what people think that disadvantaged groups are does not fit with the normative features that people are expected to have in economically unequal settings. In other words, if women and ethnic minority groups are not seen as possessing the traits expected in an unequal context (e.g., competence, dominance, and intellectual and leadership skills), it is reasonable to think that these social groups would be considered less representative of such contexts. Gender roles prescribe feminine (i.e., communal) behaviours and proscribe masculine (i.e., more dominant and competitive) ones for women. Thus, women risk not being accommodated in economically unequal contexts because their communality-oriented social role conflicts with the prevalent agentic norms of such settings [5]. In addition, economic inequality is also a reflection of other forms of discrimination, such as racism [38]. For instance, the growth of economic inequality is related to the growth of the racial income gap [39]. Furthermore, economic inequality provides a context that affects the perpetuation of norms that support social inequalities (e.g., sexism and racism [40]) in individuals’ minds.

Overall, the second aim of our research was to investigate whether perception of economic inequality in an organizational context would create implicit beliefs that the presence of women and ethnic minority group members would be lower in such a setting.

### The mediating role of perceived diversity climate

A diversity climate consists of the recognition and valuing of individual and group differences [41]. It implies a body of organizational policies and decisions that promote the acceptance of gender and ethnic minority groups. From a psychological perspective, a diversity climate reflects the organization’s attitudes towards people who are not the mainstream in organizations [42]. Hence, the construct of diversity is inherently related to the inclusion-exclusion and discrimination of women and ethnic minority groups [43, 44]. Often a White male worker is considered to be prototypical or the mainstream, particularly in domains seen as more masculine (e.g., science, technology, engineering, and mathematics [STEM] careers) that exclude people who do not belong to these social categories [45–47].

Following the same logic, a White man can be considered to be the prototypical member of an organization that is unequal (rather than equal). Thus, if the organization is unequal, people likely infer that it is less diverse. It has been proposed that economic inequality promotes social division and is associated with the impermeability of group boundaries [48]. Moreover, Caluori et al. [49] suggested that economic inequality is a powerful socio-economic variable that highlights differences between groups, diminishes intergroup contact, and heightens prejudice towards minorities. We thus expected that economic inequality would lead to a climate characterized by a low acceptance of diversity.

Furthermore, the perception of a diversity climate can significantly impact different groups’ expectations of being a part of an organization. For instance, previous research has shown that ethnic minority women, who belong to two minority groups, feel excluded from the organizational power structure [50] and that a pro-diversity climate makes people from gender and ethnic minority groups feel more strongly that they are part of an organization and, in turn, positively reflects on their job-related and group-related attitudes (e.g., job satisfaction and job performance [51] and in-group support [52]). In contrast, feeling dissimilar to teammates—at both the surface and deep levels—negatively affects feelings of inclusion in an organization for minority group members [34, 53]. At the same time, group-based disparities in dimensions such as gender, ethnicity, and even political beliefs may increase discrimination [54]. If the social dynamics presented in unequal settings are associated with social norms whose content reflects such social dynamics [12, 19], we can expect that, in economically unequal settings, people will perceive that the norm concerning diversity is weak.

Considering this body of research, the third aim of our work was to explore the role of the perception of the diversity climate in the relationship between economic inequality and the expected presence of women and ethnic minority groups. More precisely, perceptions of larger gaps in the distribution of economic resources to low- and high-status employees in an organization could lead to the perception that individuals of diverse backgrounds are also valued and treated differently within this organization. Thus, economic inequality within the organizational context could lead to the perception of an adverse diversity climate, which, in turn, could lead to estimations of a lower presence of women and ethnic minority group members in this organization, compared to an equal one. In other words, diversity climate perception could explain *why* economic inequality induces expectations of lower diversity in terms of numbers amongst employees.

## The present research

In the present paper, we investigated the effect of economic inequality in terms of (un)equal distribution of incomes and resources between the highest- and lowest-status individuals in an organizational setting. This was inspired by manipulations of economic inequality used in a general societal context (e.g., [55]). We expected that, in an organizational setting with high economic inequality, participants would expect a stronger presence of men (vs. women) and a greater presence of members of an ethnic majority group (vs. a minority group).

For the present paper, we manipulated economic inequality by varying the size of the gap in the distribution of financial and intangible incentives between groups of high-ranking and low-ranking employees, and measured its effects on the main dependent variable, perceived presence of minority group members, and the mediator, perceived diversity climate. More specifically, we re-examined two previous studies [56], which are combined in a pooled analysis (Studies 1a and 1b), exploring the relations we proposed. Then, to deepen the initial exploratory findings, we conducted Study 2, in which we wanted to test two hypotheses: We predicted that, in an organizational setting with a high or moderate level of economic inequality versus a setting with equality, people will infer a lower presence of women (vs. men; Hypothesis 1) and of minority ethnic group members (vs. majority ethnic members; Hypothesis 2) as a part of the workforce. Moreover, we added the exploration of perceived diversity climate as a mediator of the relationship between economic inequality and gender or ethnic minority presence. We preregistered the hypotheses, all measures, and analyses at the Open Science Framework (OSF).

## Studies 1a and 1b

This set of studies includes exploratory data collected but not reported in the context of another paper [56]. We combined two datasets, referred to as Studies 1a and 1b, and we conducted a pooled analysis.

### Method

Given that we obtained data from two different samples, we first standardized continuous variables in each database (Studies 1a and 1b) and then merged them into a single database. The scores of the continuous manipulation check and the presence of minority group members of this study are indicated as standardized values.

#### Participants and procedure

In Study 1a we collected data from 203 people on Amazon Mechanical Turk (MTurk). We excluded six participants who asked for their responses to be deleted and two who had missing values (*N* = 195; *n* = 69 women; *M*_*age*_ = 35.60 years, *SD* = 10.55). In Study 1b we collected 187 observations. Participants were psychology students from a Swiss university. We excluded two participants who did not give their informed consent and two who had missing values (*N* = 183; *n* = 153 women; *M*_*age*_ = 22.75, *SD* = 4.35). The final sample was composed of 378 participants (222 women, *M*_*age*_ = 29.38, *SD*_*age*_ = 10.38). All of them gave their informed written consent. The study was performed in accordance with the ethical standards as laid down in the 1964 Declaration of Helsinki and its later amendments or comparable ethical standards. The study was also approved by the ethical committee of the University of Geneva.

The manipulation and measures were identical in Study 1a and Study 1b except for the type of participant samples and a minor change in the information we presented in the experimental condition of low inequality concerning the size of inequality (see below).

#### Experimental manipulation of economic inequality

All participants were randomly assigned to one of three experimental conditions (High economic inequality vs. Low economic inequality vs. Economic equality). To manipulate economic inequality, we used an experimental manipulation in an organizational context [56]. Participants read a scenario about an organization with relatively large (high economic inequality condition, *n* = 133), small (low economic inequality condition, *n* = 120), or no differences (economic equality condition, *n* = 125) in salary and access to resources between the employees with the highest managerial positions and the employees with lower positions: “Imagine a large organization. In this organization, as in most large organizations, employees have different roles and functions. There are employees at higher and lower ranks. Those at the higher ranks have managerial positions, and those at lower ranks have non-managerial positions.” Then the manipulation of economic inequality was introduced. The following excerpt pertained to the high economic inequality condition of Study 1a (we present the variants for the low inequality and equality conditions in parentheses): “In this organization, employees who occupy the highest managerial positions earn about 50 times more than (low inequality: 10 times more than; equality: the same as) those who occupy the lowest positions in the organizational hierarchy. Moreover, employees in managerial positions enjoy other benefits: 15% additional vacation time (low inequality: 5% additional vacation time; equality: the same amount of vacation time) and a private 400-square-foot office (low inequality: 150-square-foot office; equality: the same office space). Employees in lower positions do not have such perks: they have the standard vacation time and shared offices. In summary, in this organization employees are rewarded differently (low inequality: differently; equality condition: similarly) depending on the roles and functions they enact in the organization. These rewards have an impact on the opportunities and services that employees have access to in their personal lives.” In Study 1b, the manipulation of economic inequality was similar, but we slightly modified the information for low economic inequality to reduce the degree of inequality. The information for the high inequality and equality conditions was the same as presented above. The information for the low inequality condition of Study 1b that differed from Study 1a was the following statement: “In this organization, managers in the highest position earn about 5 times more than those who occupy the lowest positions in the organizational hierarchy. Moreover, employees in managerial positions enjoy other benefits: 3% additional vacation time and an individual office of 15m2.”

After the experimental manipulation, we included two manipulation checks: “How much do employees with managerial positions in this organization earn in relation to other employees with non-managerial positions?” with three options, each of which reflected one of the differences reported in the manipulations (Study 1a: 1 = *50 times more*, 2 = *10 times more*, 3 = *the same;* Study 1b: 1 = *50 times more*, 2 = *5 times more*, 3 = *the same*). A second item measured the participants’ perception of the differences: “How large do you consider the economic differences to be between employees in managerial positions and non-managerial positions in this organization?” on a 5-point Likert scale ranging from 1 (*Very small differences*) to 5 (*Very large differences)*.

#### Presence of minority/majority group members

We used the statement “Based on the information on the organization, what do you think is the distribution of the following groups working in this organization?” to ask participants about their estimates of the distribution of social groups working in the organization concerning: employees’ gender (1 = *Mainly women* to 7 = *Mainly men;* Study 1a: *M* = 4.83, *SD* = 1.28; Study 1b: *M* = 4.68, *SD* = 1.23), and the ethnicity of employees (1 = *Mainly White majority employees* to 7 = *Mainly ethnic minority employees;* Study 1a: *M* = 3.96, *SD* = 1.58; Study 1b: *M* = 3.11, *SD* = 1.32). Values were standardized to run the analyses.

*Sociodemographic information*. Finally, we asked participants to provide sociodemographic information, such as gender, age, educational attainment, and income level.

#### Statistical analyses

First, to assess whether participants remembered the information on economic differences in the experimental condition they were assigned, we looked at cross tabulations. Second, we ran an ANCOVA to check whether the experimental manipulation worked. In this ANCOVA we included the experimental manipulation (economic inequality) as a between-groups variable and the standardized continuous manipulation check as a dependent variable. We controlled for study (Study 1a vs. Study 1b) to test whether the effect of the experimental manipulation remained stable across the studies. In the ANCOVA, we also ran Bonferroni-adjusted pairwise comparisons to compare the means between the three experimental conditions. Then, in order to examine the effect of economic inequality on the dependent variables, we ran a MANCOVA in which economic inequality was included as a between-groups factor and the perceived presence of minority or majority groups for each social category (gender and ethnicity) as dependent variables. We added the study variable (Study 1a vs. Study 1b) as an independent variable to control for it and for the Economic Inequality × Study interaction. We also performed Bonferroni-adjusted pairwise comparisons in the MANCOVA.

### Results

#### Manipulation checks

Cross-tables revealed that most participants remembered the salary indicated in the experimental condition that they were assigned to (82% in high economic inequality, 95% in low economic inequality, and 77.6% in economic equality).

The results of the ANCOVA revealed that the economic inequality manipulation worked: those participants assigned to the high economic inequality condition considered economic differences between the highest-ranking managerial employees and the lowest-ranking non-managerial employees to be higher (*M* = 0.60, *SD* = 0.55) than in the lower inequality (*M* = 0.35, *SD* = 0.56; *M*_*D*_ = 0.25, *p* = .016) and equality conditions (*M* = -0.97, *SD* = 0.97, *M*_*D*_ = 1.58, *p* < .001) and higher in the lower inequality than in the equality condition *(M*_*D*_ = 1.32, *p* < .001), *F*(2, 374) = 175.84, *p* < .001, η_p_2 = .48. We did not observe an effect of the study covariate, *F*(1, 374) = 1.52, *p* = .218, η_p_2 < .01, nor an interaction between economic inequality and the covariate, *F*(2, 374) = 0.80, *p* = .452, η_p_2 < .01.

#### Testing the effect of economic inequality on the presence of minority/majority group members

The MANCOVA analysis revealed that the manipulation had a multivariate effect, Wilks’s lambda = .78, *F*(4, 746) = 25.16, *p* < .001, η_p_2 = .12. We did not observe an effect of the covariate on any dependent variable. However, we observed a multivariate Economic Inequality × Study interaction, Wilks’s lambda = .97, *F*(4, 742) = 2.74, *p* = .028, η_p_2 = .02. Below we present the effect of economic inequality on each dependent variable (gender and ethnicity) and then the interaction effect on each variable. Table 1 reports overall descriptive statistics for the main dependent variables, and Table 2 for Studies 1a and 1b separately. Additional analysis including sociodemographic variables as covariates showed similar results.

**Table 1:** Means and standard deviations of perceived presence of minority/majority groups in terms of gender and ethnicity in Study 1a and 1b (pooled).

	Higher EI (*n* = 133)	Lower EI (*n* = 120)	Economic Equality (*n* = 125)
	*M* (*SD*)	*M* (*SD*)	*M* (*SD*)
Gender	0.27 (0.99)	0.29 (0.80)	-0.56 (0.94)
Ethnicity	-0.35 (1.00)	-0.10 (0.97)	0.46 (0.88)

*Note*. Measures were standardized. Gender: Higher rates indicate lower perception of the presence of minority group members within the organization. Ethnicity: Higher rates indicate more perception of the presence of minority group members within the organization. EI: Economic inequality.

**Table 2:** Means and standard deviations of perceived presence of minority/majority groups in terms of gender and ethnicity in Study 1a and 1b (separately).

	Study 1a (American Sample)			Study 1b (Swiss Sample)		
	High EI (*n* = 74)	Low EI (*n* = 62)	Economic Equality (*n* = 59)	High EI (*n* = 59)	Low EI (*n* = 58)	Economic Equality (*n* = 66)
	*M* (*SD*)	*M* (*SD*)	*M* (*SD*)	*M* (*SD*)	*M* (*SD*)	*M* (*SD*)
Gender	0.20 (0.96)	0.16 (0.88)	-0.42 (1.04)	0.35 (1.02)	0.42 (0.68)	-0.69 (0.84)
Ethnicity	-0.38 (0.94)	0.10 (0.97)	0.98 (0.94)	-0.31 (1.07)	-0.30 (0.86)	0.53 (0.82)

*Note*. Measures were standardized. Higher rates indicate lower perception of the presence of minority group members within the organization. Ethnicity: Higher rates indicate more perception of the presence of minority group members within the organization. EI: Economic inequality.

*The gender dimension*. Univariate results showed a main effect of economic inequality on the gender dimension, *F*(2, 377) = 35.17, *p* < .001, η_p_2 = .16. Bonferroni-adjusted pairwise comparisons showed that, in the organizations with high and low economic inequality, participants indicated that employees would be more likely to be men compared to the equality context (*M*_*D*_ = 0.83, 95% CI [0.56, 1.11], *p* < .001; and *M*_*D*_ = 0.85, 95% CI [0.57, 1.14], *p* < .001, respectively). No difference occurred between the high and the low economic inequality conditions (*M*_*D*_ = -0.02, 95% CI [-0.30, 0.26], *p* >.999). Overall, in both unequal contexts, people considered female employees to make up a smaller portion of the workforce.

*The ethnicity dimension*. Univariate results further showed a main effect of economic inequality on the ethnicity dimension, *F*(2, 374) = 24.82, *p* < .001, η_p_2 = .12. Bonferroni-adjusted pairwise comparisons revealed that, in the high and low economic inequality conditions, participants indicated that employees would more likely belong to the White majority (rather than an ethnic minority) compared to the economic equality condition (*M*_*D*_ = -0.80, 95% CI = [-1.08, -0.52], *p* < .001; *M*_*D*_ = -0.56, 95% CI = [-0.84, -0.27], *p* < .001, respectively). We did not observe a difference between the high and low economic inequality conditions, *M*_*D*_ = -0.25, 95% CI = [-0.53, 0.04], *p* = .116. Overall, people were less likely to expect ethnic minorities to be employees in unequal organizations.

*Economic Inequality* × *Study interaction*. Looking at univariate effects, we found that an interaction occurred only for the ethnicity dimension, *F*(2, 372) = 3.06, *p* = .048, η_p_2 = .02. Pairwise comparison showed an effect for the low economic inequality condition, *F*(1, 372) = 5.29, *p* = .022, η_p_2 = .01, where American participants (Study 1a) indicated that they expected a stronger presence of White majority employees in the organization than Swiss participants did (Study 1b, *M*_*D*_ = 0.39, 95% CI [0.57, 0.73], *p* = .022).

### Discussion

The results of this first exploratory set of studies suggest that economic inequality in an organization, independent of the level of inequality (low or high), led participants to expect that it would coincide with a lower presence of minority group members (women and ethnic minorities) amongst the employees of the organization in comparison with an equal organization, which was perceived as having a higher presence of women and ethnic minority individuals. Our main interest was to compare an inequality context with an equality context. However, it is of interest to point to the absence of differences in the representation of minority groups in these two unequal contexts. We believe that both represent an unequal structure that supports socioeconomic hierarchy and that this aspect is given greater attention than its exact size, particularly in relatively equal societies such as the US and Switzerland.

We had no prior expectations as concerns cultural differences between the two samples and no direct sample effect was revealed (Study 1a vs. Study 1b). However, the effect of economic inequality on the ethnicity dimension varied by sample. Notably, we observed that, in the lower economic inequality condition, American participants expected a stronger representation of White employees than Swiss participants did. Following the Black Lives Matter movement of recent years, race seems to be a much more strongly embedded and accessible social category for Americans than ethnicity for Swiss people.

Overall, the presented results derive from experiments which were designed with a different aim and thus were not intended to test specific predictions. Despite this limitation, the findings provide some preliminary insights into the potential links between economic inequality and the perceptions of minority representation in the workforce, and contributed to the formulation of our Hypothesis 1.

## Study 2

We sought to replicate the effect of economic inequality on the perceived presence of minority and majority group members in a new study that was specifically designed to test our hypotheses using the same experimental design as in Study 1a and Study 1b. Importantly, Study 2 was thus preregistered and planned for a large sample size. We predicted that minority groups (women in Hypothesis 1 and ethnic groups in Hypothesis 2) should be less likely expected to be employees in an organization described as distributing economic resources in unequal ways across employees of different hierarchical positions than in an organization where distribution is equal.

A novelty of Study 2 was that we planned to examine whether knowledge about the presence or absence of inequality in an organization would impact the perception of the diversity culture of the organization. The existing literature suggests that organizational wage systems are a useful clue about organizational culture [57, 58]. Diversity climate is conceptualized as an organizational climate that reflects openness to and appreciation of individual differences [41]. Research has revealed that this psychological dimension, the perception of a positive diversity climate, is related to increased workgroup involvement [59] and a reduction of aggression and diversity-related conflict [41, 60]. Thus, taking this study a step forward, we included a measure of perceived diversity climate and explored the impact of economic inequality on diversity climate perceptions and its consequent effect on expectations of the presence of minority and majority group members.

### Method and procedure

#### Participants

We calculated a priori sample size analysis using G*Power [61]. To detect a medium effect size (*f* = .20) using a statistical power of .80 and alpha error probability of 5%, the optimal sample size would be 246 participants. We wanted to recruit at least 100 participants per experimental condition. Following this rationale, we preregistered to collect a minimum of 300 and a maximum of 400 valid observations. Due to a technical problem on MTurk, we ended up having a total of 500 responses. Following the preregistered exclusion criteria, we excluded 51 participants because they did not indicate that they have at least a good proficiency in English. The final sample was composed of 449 participants (210 women, *M*_*age*_ = 40.62, *SD* = 11.73). Before starting the study block, all participants gave their informed written consent. This study was performed in accordance with the ethical standards as laid down in the 1964 Declaration of Helsinki and its later amendments or comparable ethical standards. It was also approved by the ethical committee of the University of Geneva.

#### Experimental manipulation of economic inequality

We used the same vignettes of organizations for the experimental manipulation of economic inequality as in Study 1b. Participants were randomly assigned to one of the three experimental conditions (High economic inequality vs. Low economic inequality vs. Economic equality). As a result, 124 participants were assigned to the high inequality condition, 166 participants to the low inequality condition, and 159 participants to the equality condition. We included the same two manipulation checks as in Study 1b.

#### Presence of minority/majority group members

We used the same statements as in Study 1a and Study 1b as dependent variables. Unlike the first set of studies, in Study 2 the answer format for employees’ ethnicity was presented as 1 = *Mainly ethnic minority employees* and 7 = *Mainly White majority employees* (*M* = 4.71; *SD* = 1.35), with the aim to indicate that lower scores represent the perceived presence of minority groups and higher scores represent the perceived presence of majority groups. The answer format for employees’ gender was the same as in the previous studies (*M* = 4.68; *SD* = 1.30).

#### Perceived diversity climate

Five diversity items from Campbell Organizational Survey (COS; [62]) assessed participants’ opinions about organizational culture in the organization presented. We replaced “our organization” with “the organization” in each item because we were evaluating the perception of the culture of the fictitious organization in the vignette. We introduced items asking participants to think about how they believed that the organization handled diversity (such as different gender, ethnicity, age, and political orientation, among other things). Next, participants completed the COS. Example items were “The organization has a diverse membership, including people with many varying personal characteristics and background” and “The members of the organization treat people fairly, no matter what their gender, race or age” (7-point Likert scale, 1 = *Strongly disagree*, 7 = *Strongly agree*, F061 = .94; *M* = 4.43, *SD* = 1.53). Higher scores mean greater perceived diversity.

*Sociodemographic information*. Like in Studies 1a and 1b, we asked participants to provide sociodemographic information (i.e., gender, age, educational attainment, income level, and political orientation).

#### Statistical analyses: Preregistered analytical strategy

We followed a similar analytical strategy as in Studies 1a and 1b. Firstly, we looked at cross tabulations. Then, to check whether the experimental manipulation worked, we conducted an ANOVA using the same criteria as in Studies 1a and 1b. Later, to test the preregistered hypotheses, we planned to conduct a MANOVA including economic inequality as a between-groups factor and the perceived presence of minority/majority groups for each social category (gender and ethnicity) as dependent variables. We also planned to run Bonferroni-adjusted pairwise comparisons to compare the means between our three experimental conditions.

### Results

#### Manipulation checks

First, cross-tables revealed that most participants remembered the salary difference indicated in the experimental condition that they were assigned to (87.1% in high economic inequality, 96.4% in low economic inequality, and 93.7% in economic equality). Second, the ANOVA revealed that the manipulation worked: participants assigned to the high inequality condition considered economic differences between the highest-ranking managerial employees and the lowest-ranking non-managerial employees to be higher (*M* = 4.67, *SD* = 0.57) than in the low inequality (*M* = 3.86, *SD* = 0.89; *M*_*D*_ = 0.81, *p* < .001) and equality conditions (*M* = 1.45, *SD* = 0.98; *M*_*D*_ = 3.22, *p* < .001); differences between the lower inequality and equality conditions were also significant (*M*_*D*_ = 2.41, *p* < .001), *F*(2, 446) = 574.43, *p* < .001, η_p_2 = 0.72.

#### Hypothesis testing: The effect of economic inequality on the presence of minority/majority group members

The MANOVA revealed a multivariate effect of manipulation, Wilks’s lambda = .79, *F*(4, 890) = 28.30, *p* < .001, η_p_2 = .11. Table 3 reports the descriptive statistics. As planned, we conducted Bonferroni post-hoc tests to analyze differences between the three experimental conditions. Analysis including sociodemographic variables as covariates showed similar results.

**Table 3:** Means and standard deviations of perceived presence of minority/majority groups in terms of gender and ethnicity in Study 2.

	High EI (*n* = 124)	Low EI (*n* = 166)	Economic Equality (*n* = 159)
	*M* (*SD*)	*M* (*SD*)	*M* (*SD*)
Gender	5.10 (1.20)	5.10 (1.14)	3.92 (1.17)
Ethnicity	4.90 (1.35)	5.20 (1.11)	4.04 (1.31)
Diversity Climate	3.61 (1.45)	4.08 (1.34)	5.42 (1.53)

*Note*. Answer format was a 7-point scale. EI: Economic inequality.

*The gender dimension*. Univariate results showed an effect of economic inequality on distributions of employees’ gender, *F*(2, 446) = 51.77, *p* < .001, η_p_2 = .19. Bonferroni-adjusted post-hoc tests showed that, in the organizations with high and low economic inequality, individuals expected a stronger presence of men (vs. women) compared to the equality context, *M*_*D*_ = 1.18, 95% CI [0.84, 1.52], *p* < .001; and *M*_*D*_ = 1.17, 95% CI [0.86, 1.48], *p* < .001, respectively. No difference occurred between high and low economic inequality conditions, *M*_*D*_ = 0.01, 95% CI [-0.33, 0.34], *p* > .999. The results support Hypothesis 1 by showing that lower presence of women is expected by participants exposed to organizations distributing economic resources unequally, independent of the size of the inequality.

*The ethnicity dimension*. We found an effect of economic inequality on the expected distribution of majority and minority employees according to their ethnicity, *F*(2, 446) = 36.52, *p* < .001, η_p_2 = .14. Bonferroni-adjusted post-hoc tests revealed that, in the conditions of high and low inequality (*p* > .05), participants expected more White majority individuals compared to the equality condition, *M*_*D*_ = 0.85, 95% CI [0.49, 1.21], *p* < .001; *M*_*D*_ = 1.15, 95% CI [0.82, 1.49], *p* < .001, respectively. We did not find a difference between high and low economic inequality conditions, *M*_*D*_ = -0.30, 95% CI [-0.66, 0.05], *p* = .125. Overall, our Hypothesis 2 was supported, as participants looking at unequal organizations inferred a lower presence of ethnic minorities as employees, independent of the size of the inequality.

#### The effect of economic inequality on perceived diversity climate

We first explored whether perceived economic inequality affected perceived diversity climate. A one-way ANOVA on perceived diversity climate revealed an effect of our between-group predictor economic inequality, *F*(2, 446) = 73.03, *p* < .001, η_p_2 = .25. Bonferroni-adjusted post-hoc tests revealed that, in the higher inequality condition, participants rated the climate as less diverse than in the low inequality (*M*_*D*_ = -0.47, 95% CI [-0.85, -0.09], *p* < .001) and equality conditions (*M*_*D*_ = -1.81, 95% CI [-2.19, -1.42], *p* < .001). Moreover, the low inequality condition was also perceived as less diverse than the equality condition (*M*_*D*_ = -1.34, 95% CI [-1.70, -0.97], *p* < .001).

#### The mediator role of perceived diversity climate

We then explored whether the perceived diversity climate mediated the negative relationship between economic inequality (vs. equality) and the presence of minority group members. We computed a global score for the presence of minority group members (the mean score of gender and ethnicity distribution ratings) for our outcome variable. We performed a mediation analysis with the PROCESS macro for SPSS (Model 4) using bias-corrected bootstrapping for 10,000 resamples and a 95% confidence interval [63]. The predictor perceived economic inequality was introduced as gradual contrast C1 (coded higher economic inequality = 1, lower economic inequality = 0, equality = -1), and its orthogonal residual contrast C2 (coded higher economic inequality and equality = -1, lower economic inequality = 2) was controlled for. The C1 contrast tests the difference between the high inequality and equality conditions. The C2 contrast verifies whether the low inequality condition is situated in-between by testing the comparison of the low inequality condition versus the high inequality and equality conditions taken together. To support a gradual effect, contrast C1 should be significant, but not the residual C2.

**Fig 1:**
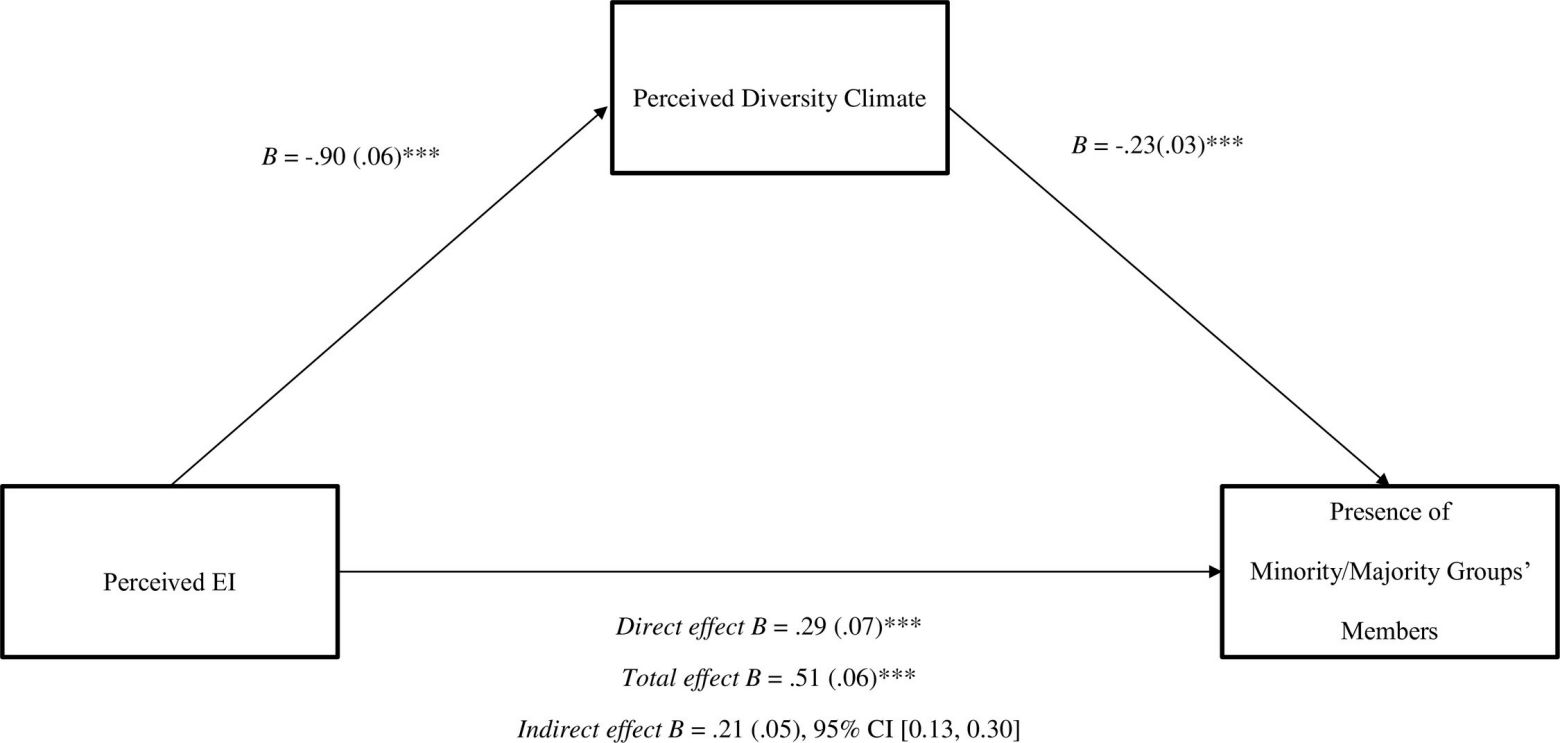
The indirect effect of perceived economic inequality (contrast C1), mediated by perceived diversity climate, on the presence of minority/majority group members. Process Model 4 of Hayes [63]. Perceived EI as a gradual effect (C1) and coded as high EI = 1, low EI = 0, equality = -1. Orthogonal residual contrast (C2) coded as high EI and equality = -1, low EI = 2. Numbers indicate unstandardized coefficients. ***p < .001.

Results revealed an indirect effect of perceived economic inequality (C1) on the presence of minority group members, *IE* = .21 (.05), 95% CI [0.13, 0.30], and a non-significant residual contrast C2. The indirect effect showed that the more unequal the organization was, the more it was perceived that the organization was composed of mainly men and White individuals through perceived diversity climate (see Fig 1).

### Discussion

Study 2 tested and found support for our two main hypotheses. In organizational contexts that distributed resources unequally between low- and high-ranking employees, people expected a less diverse organizational make-up to the detriment of women (Hypothesis 1) and members of ethnic minorities (Hypothesis 2) in comparison to an organizational context with equal distribution of resources. Moreover, in line with Study 1, participants expected a higher proportion of majority individuals than minority individuals (in terms of gender and ethnicity) in the unequal contexts. However, it is of interest that, in contrast to the previous set of studies, the mean scores indicated that participants did not perceive more women than men or more ethnic minority members than majority members within the equality condition, but they perceived there to be more women and ethnic minority members than in the unequal conditions.

Study 2 further explored the role of diversity climate expectations, our third investigatory aim, as a potential mechanism explaining the diminished expectations of a diverse make-up in unequal contexts. Our findings suggest that the stronger economic inequality was perceived to be, the less pro-diversity organizational culture was perceived to be. Exploratory mediation analysis then established that the decreased perceptions of a positive diversity climate in unequal contexts indeed predicted the expectation of lower presence of minority group members. This evidence suggests that economically unequal contexts promote intergroup relations that are less respectful of minority groups, and therefore people believe fewer members from these groups will be present, either because they do not feel welcome and opt out or because discriminatory practices cause them to be overlooked in recruitment and promotions [64]. Overall, this provides indirect evidence about the detrimental effect that economic inequality may have on intergroup relations.

Finally, despite the finding that perceived distribution of minority and majority employees did not differ between high and low levels of economic inequality, we observed that participants perceived the climate as less tolerant to diversity in the high economic inequality condition than in the low one. These results raise questions regarding the different impact of economic inequality on certain social psychological outcomes (i.e., the perceived presence of minority groups and the perceived diversity climate).

## General discussion

We expanded on prior research about the effects of perceived economic inequality on perceptions of individuals and groups by investigating such tendencies in an organizational context and by analyzing its effects on expectations that an environment will be socially inclusive versus exclusive and the consequent representation of minority/majority group members. One set of two exploratory studies (Study 1a and Study 1b) and a preregistered study (Study 2) provided evidence that contexts considered unequal (i.e., high and low economic inequality) led people to expect the organizational climate to be less tolerant towards minority groups, leading to a lower expected representation of these groups. Our results are in line with previous studies that have shown that economically unequal contexts may lead to a descriptive norm dominated by masculinity (vs. femininity) [5] and that they reflect ethnic inequalities [38].

One exploratory result that emerged from our research was a gradual effect showing that higher economic inequality was perceived as supporting a less diverse culture not only in comparison to the equal setting but also relatively to the lower unequal context. This result appears to indicate that, although the proportion of men and White employees was not perceived as different in high vs. low inequality settings, the diversity culture is. Previous research has shown that, the higher diversity is perceived to be, the more people perceive similarities in competence and warmth between different ethnic groups and the more positively minorities (e.g., immigrants) are viewed [65]. According to these authors, a positive diversity climate may help attenuate exaggerated stereotypes, thereby reducing perceived threat from an out-group and consequently creating more positive intergroup relationships. Based on these observations and our own findings, future research may explore how stereotypes of gender and ethnic groups differ depending on perceived economic inequality and their association with diversity climate.

An explanation of our main results could be based on the availability heuristic. When an availability heuristic is active, perceivers evaluate a case’s likelihood based on how quickly other similar examples come to their mind [66]. At first glance, one could assume that organizational contexts automatically activate the male/White employee prototype, making these majority categories more accessible examples in organizations [67, 68]. However, we found variations in the presence of minority/majority group members across experimental conditions. Thus, this suggests that people retrieved some exemplars or memories that led them to link economic inequality (not organizational contexts) with a more homogeneous social setting and with the higher presence of advantaged social groups such as men and White people. That is, economic inequality seems to be a factor that might modulate the ease of recall related to gender and ethnicity hierarchy.

Another explanation of the matching of higher economic inequality with a higher proportion of men and White employees could be that people viewed such hierarchical systems as more competitive, creating differences in power, and hierarchy-enhancing in nature [3, 20]. Our experimental conditions all included status differences that we held constant across conditions. However, what changed across conditions is the economic power that employees would have in their lives following the unequal distribution between high and low status employees. In accordance with social dominance theory [69], participants could have inferred that employees who adhere to hierarchy-enhancing ideologies and are motivated by incentives (as a means of acquiring economic power) would more likely want to work in such unequal systems (hoping to be amongst the high earners). At the same time, organizations themselves would seek out employees who adhere to hierarchy-enhancing ideologies, and would further socialize them in this ideology. Majority groups people (men and White people) have been shown to more strongly adhere to hierarchy-promoting ideologies and have higher scores of racism and sexism [70]. Overall, such dynamics could have led to a perception of a less diverse climate and a higher representation of majority employees. In contrast, equal contexts are associated with hierarchy-attenuating ideologies typically found in women and minority groups [71, 72].

### Practical implications and future research

If social norms and inferences affect individuals’ behaviour and attitudes [1], it seems worthwhile to explore real organizational settings with different degrees of economic inequality and make a comparison between them and their diversity culture and, going beyond that, to examine whether other factors are affected, such as employees’ performance [57]. Moreover, because perception of higher economic inequality emphasizes feeling different from others, it would be of interest to examine this in more depth. For instance, in those organizations with larger economic differences between employees, the employees’ feeling of being dissimilar to their colleagues could be greater in comparison with the feeling of employees in other, more egalitarian organizations and the effects of this feeling on their psychological well-being [73] according to their organizational status.

It is important to note that the participants’ attitudes towards their own in-group and out-group in terms of gender or ethnicity could have influenced their responses. However, we did not have information on participants’ intergroup attitudes. According to Bianchi [74], varying economic conditions may affect racial attitudes, but we also believe that it may affect gender attitudes in the sense that it is important to examine how advantaged and disadvantaged groups’ members infer the presence of in-group and out-group members in specific socioeconomic conditions. Further research could consider people’s attitudes towards majority and minority groups and examine whether these attitudes moderate the relationship between perceived economic inequality and perceived presence of minority/majority group members.

## Conclusions

The current research contributes to the literature about economic inequality and its psychosocial effects. Specifically, we shed light on three questions. First, we examined whether people’s expectations change in response to perceived economic inequality in organizational contexts. To do that, we used an experimental manipulation based on economic differences in an organization, which allowed us to focus on the effect of perceived economic inequality in a context different from a larger societal context. This is a novelty, as much of the research on economic inequality has focused on society as a whole. Second, perceived economic inequality affects people’s perception of who works in an organization. Our results revealed that unequal organizational environments led to more inferences about a higher presence of men and White people—and then a lower presence of women and members of ethnic minority groups—as part of the workforce. Third, we intended to explore the mechanism explaining such inferences. We observed that the organizational setting with higher inequality was considered less supportive of a diverse culture, which mediated the relationship between economic inequality and perceived presence of minority group members. Overall, this evidence enhances understanding of the prominent role of perceived economic inequality on psychosocial factors related to intergroup relations.
